# Compliance with anthelmintic treatment in the neglected tropical diseases control programmes: a systematic review

**DOI:** 10.1186/s13071-016-1311-1

**Published:** 2016-01-27

**Authors:** Kathryn V. Shuford, Hugo C. Turner, Roy M. Anderson

**Affiliations:** London Centre for Neglected Tropical Disease Research, London, UK; Department of Infectious Disease Epidemiology, School of Public Health, Faculty of Medicine, St Marys Campus, Imperial College London, Norfolk Place, London, W2 1PG UK

**Keywords:** Compliance, Coverage, Systematic non-compliance, NTDs, Helminths, Preventive chemotherapy, Mass drug administration, Elimination, Systematic review

## Abstract

Preventive chemotherapy (PCT) programmes are used to control five of the highest burden neglected tropical diseases (NTDs): soil-transmitted helminth infections (hookworm, ascariasis, and trichuriasis), lymphatic filariasis, schistosomiasis, onchocerciasis, and trachoma. Over the past decade, new resource commitments for the NTDs have enabled such programmes to intensify their control efforts, and for some diseases, to shift from goals of morbidity control to the interruption of transmission and elimination. To successfully eliminate the parasite reservoir, these programmes will undoubtedly require prolonged, high treatment coverage. However, it is important to consider that even when coverage levels reach an acceptable proportion of the target population, there may be a considerable gap between coverage (those who receive the drug) and compliance (those who actually consume the drug)—a topic of fundamental and perhaps underestimated importance. We conducted a systematic review of published literature that investigated compliance to PCT programmes for NTD control and elimination. Databases searched included PubMed/Medline, Web of Knowledge (including Web of Science), OVID, and Scopus. Data were collected on compliance rates, reasons for non-compliance, as well as the heterogeneity of compliance definitions and calculations across programmes and studies. A total of 112 studies were selected for inclusion. The findings of the review revealed substantial heterogeneity across compliance terms and definitions; an imbalance of available studies for particular disease areas and countries; and finally, a lack of longitudinal compliance studies to properly investigate the role of systematic non-compliance. The lack of consistency among reporting of compliance data can result in under- or over-estimating compliance in a population, and therefore has serious implications for setting and reaching elimination targets. Reframing of the guidelines on compliance definitions coupled with an urgent call for longitudinal research in systematic non-compliance should be essential elements in the programmatic shift from control to elimination.

**Electronic supplementary material:**

The online version of this article (doi:10.1186/s13071-016-1311-1) contains supplementary material, which is available to authorized users.

## Introduction

The neglected tropical diseases (NTDs) affect over 1.4 billion of the world’s poorest people—including 800 million children [1, 2]. These diseases can cause long-term disability and are estimated to account for over 500,000 deaths per year [1]. Infection can lead to malnutrition, cognitive impairment, and poor school attendance—effectively trapping individuals in a cycle of poverty and associated disease [2, 3].

Preventive chemotherapy (PCT) programmes are used to control five of the highest burden NTDs: soil-transmitted helminth infections (STH) including hookworm, ascariasis, and trichuriasis species, lymphatic filariasis (LF), schistosomiasis (SCH), onchocerciasis or river blindness (RB), and trachoma. Preventive chemotherapy, often provided through mass drug administration (MDA), is given regardless of whether or not the individuals are infected. It can be aimed at a particular occupational or age group that may represent those most at-risk of heavy infection and concomitant morbidity (often children). The level and mechanism of PCT delivery may depend on local policies, resources, and disease endemicity [3, 4].

Generally, the drugs are given as a single dose at regular intervals, usually annually or bi-annually, again depending on national policies, transmission dynamics in a defined setting and endemicity [4]. Co-endemicity and polyparasitism are common in many populations; in such cases, there are advantages to integrating multiple PCT-dependent interventions, which can include cost savings of up to 50 % [2, 4–6].

The last decade has seen a period of intensifying treatment coverage and new resource commitments for NTD control. The spirit of this expanded effort is captured in the London Declaration of January 2012, where many philanthropic organisations, pharmaceutical companies (who make drug donations), as well as government and international agencies pledged further commitments to achieve new goals of control and/or elimination of ten of the highest burden NTDs by the year 2020. These goals were inspired by the World Health Organization’s (WHO) 2020 road map for accelerating work to overcome the global burden of NTDs [7]. This has enabled programmes to intensify their control efforts, and for some diseases, the goals have now shifted from morbidity control to the interruption of transmission and elimination of the parasite reservoir altogether [4, 7].

**Fig. 1:**
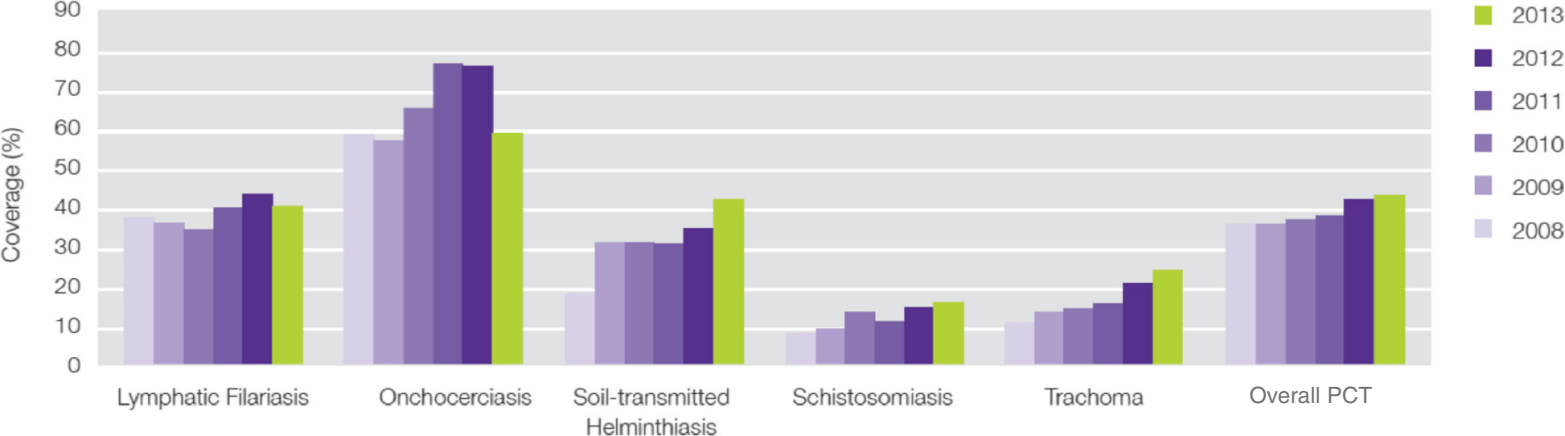
Yearly coverage of preventive chemotherapy in the NTDs. The graph shows PCT coverage from 2008 to 2013 by disease and total coverage (indicated by ‘PCT’). Adapted from [11]

The success of elimination programmes may be influenced by a wide range of factors such as initial levels of endemicity and transmission intensities in the local environment; heterogeneities of host exposure, susceptibility, and predisposition; vector/intermediate host competence, treatment frequency, duration, coverage and compliance, among others [2, 4, 8]. In particular, coverage is a commonly reported metric and a key determinant for achieving programme targets [9, 10]. Figure 1 shows annual PCT coverage by disease from 2008 to 2013 [11]. Successful elimination programmes will undoubtedly require prolonged, optimised treatment coverage—a claim widely supported by findings in the mathematical modelling literature on the impact of PCT on parasite transmission dynamics [8, 12–14]. Between 2011 and 2013, over 700 million people received PCT annually—representing only about 37 % of the 1.902 billion people across 125 countries who require annual treatment [3, 15]; the focus is now on scaling up NTD programme coverage [16].

However, it is important to note that even when coverage levels reach a significant fraction of the target population, there may be a considerable gap between coverage and compliance—a topic of fundamental and perhaps underestimated importance. Babu and Babu [17] recently explored this topic in a comprehensive review of PCT programmes for LF in India. The authors point out that in the context of India’s MDA Programme to Eliminate Lymphatic Filariasis (PELF), ‘reported coverage’ (total population to whom the drugs are delivered) is not an accurate reflection of the actual number of people consuming the drug. Consequently, ‘coverage’ and ‘compliance’ were devised as two distinct indices of PCT—the former referring to the proportion of eligible people who received tablets, and ‘compliance’ referring to the proportion of eligible people who actually ingested the tablets. Similarly, Brieger et al. explain that while reports of PCT coverage for onchocerciasis are encouraging, these rates may not give the full picture of the success of PCT due to the groups of individuals who systematically fail to comply over the years of the programme [18]. If a significant proportion of a community continually fails to comply with treatment, a portion of the parasite reservoir remains untreated and thus allows potential recrudescence via continued transmission, thereby reducing the programme’s chances at achieving elimination [17, 19]. The implications could be even more severe if one considers, for example, the possibility that those who do not comply with treatment are heavily infected individuals who experience side effects on first treatment, they may continue to be non-compliant in future rounds. Although it has not been quantified, the contribution of non-compliance (and to an even larger extent, persistent non-compliance) to transmission and its threat to elimination is likely substantial [10]. Drug compliance is, arguably, the best indicator of how well PCT is implemented; some studies have estimated 80 % to be an adequate level of drug compliance [20, 21].

The heterogeneity of language used to describe the concept of compliance is of fundamental importance to both the evaluation of PCT and for reaching targets and achieving elimination goals. Many publications have discussed the inconsistencies in the reporting of compliance rates and the range of language used to describe the concept of “medication-taking” [17, 22–28]. Two of the most commonly used and commonly debated terms are ‘compliance’ and ‘adherence’. Many argue that ‘compliance’, and in some cases both terms [27], may exaggerate the role of the physician and his/her control over the process of taking medication [22, 23, 28], and that none of these terms accurately represent patients’ motivations for choosing to take or not to take their medication [27]. Table 1 summarises some of the most common terms employed throughout the literature. These issues will be discussed further in subsequent sections of the review.

**Table 1:** Relevant terms/definitions used in medication-taking literature and sources

Term	Definition	References and comments
Compliance	Defined as *“the extent to which a person’s behaviour – taking medication, following a diet, and/or executing lifestyle changes—coincides with medical or health advice”*	[22, 28]; some argue it suggests the patient has a passive role in following provider’s orders
Adherence	Defined as “*the extent to which a person’s behaviour – taking medication, following a diet, and/or executing lifestyle changes, corresponds with agreed recommendations from a health care provider*”	[62] merged definition based on Haynes [63] and Rand [64]; preferred by some as they argue it suggests the patient is a more active partner in the decision-making
Concordance	Defined as *“a negotiated, shared agreement between clinician and patient concerning treatment regimens, outcomes, and behaviours; a more cooperative relationship than those based on issues of compliance”*	[65, 66] Royal Pharmaceutical Society of Great Britain’s working Party on medicine-taking; relatively recent term introduced by [66] to replace compliance
Other terms used to describe medication-taking:	Patient participation, acceptance, uptake, consumption	

Quantifying and understanding reasons behind compliance/non-compliance with PCT is of paramount importance, now more than ever, as NTD programme goals continue to shift from control to elimination. There is considerable literature on PCT coverage, compliance and associated factors; however, there are few comprehensive reviews on compliance rates thus far ([17, 25]), and none which address multiple NTDs and delivery methods (community- and school-based). The objective of this paper is to systematically review the published information on compliance with PCT, with a particular focus on longitudinal studies in order to investigate systematic non-compliance, for lymphatic filariasis, soil-transmitted helminths (hookworm, ascariasis, and trichuriasis), schistosomiasis, onchocerciasis, and trachoma.

## Review

We conducted a systematic review of published literature that investigated compliance to PCT programmes for NTD control and elimination. Data were collected on compliance rates, reasons for non-compliance, as well as the heterogeneity of compliance definitions and calculations across programmes and studies. The authors followed the guidelines under the PRISMA Statement for Reporting Systematic Reviews and Meta-Analysis. The completed PRISMA checklist is available in Additional file 1.

### Criteria for selection of studies

Papers investigating compliance to community- and/or school-based PCT programmes that reported primary compliance data were eligible for inclusion. If these initial criteria were met, we also included quantitative data on the reasons for non-compliance and/or the presence of statistically supported compliance-related factors (only if clear that factors were assessed in their relation to compliance, not coverage). Studies reporting data on coverage were excluded unless the metric used was explicitly defined as capturing those who complied/ingested the drug, not simply having received it (e.g. studies using terms ‘drug coverage’ or ‘treatment coverage’ were often included as these are terms for compliance used by the Global Programme for Elimination of Lymphatic Filariasis (GPELF) and in most cases could be interpreted as referring to those who ingested the drug). Studies evaluating alternative interventions such as increased sensitisation/education or different recruitment strategies to improve aspects of PCT were excluded, unless the rates for the control arm (standard PCT-only) were reported separately. Due to the number of overall studies, purely qualitative studies were excluded, as were studies evaluating selective PCT (screening followed by treatment of infected individuals only). The set of LF publications from India was primarily based on the collection included in Babu and Babu’s 2013 review (this was assumed to be comprehensive); in addition, LF studies based in others countries during this time period, as well as studies completed after this publication were considered for inclusion. Case control studies which purposively selected compliant and non-compliant groups for further analysis were excluded, as were studies which elicited general compliance data over a non-specified time period (e.g. ‘have you ever taken drugs for [LF, onchocerciasis, etc.] before?’). In addition, some studies were excluded on the basis of other study design factors such as extremely small sample size or requirements to pay a fee for treatment.

### Literature search and selection of articles

A systematic search of databases including PubMed/Medline, Web of Knowledge (including Web of Science), OVID, and Scopus was performed using the following key words (and possible variants of the terms including alternate species names): *soil-transmitted helminths, helminths, ascaris, hookworm, trichuris, onchocerciasis, river blindness, schistosomiasis, bilharzia, elephantiasis, lymphatic filariasis, trachoma AND compliance, non-compliance, non-compliers, adherence, non-adherence, refusal, medication adherence*. The full search strategy is included in Additional file 2. Papers published up to 21^st^ April 2015 and available in English or French were considered.

### Selection and extraction of data

**Fig. 2:**
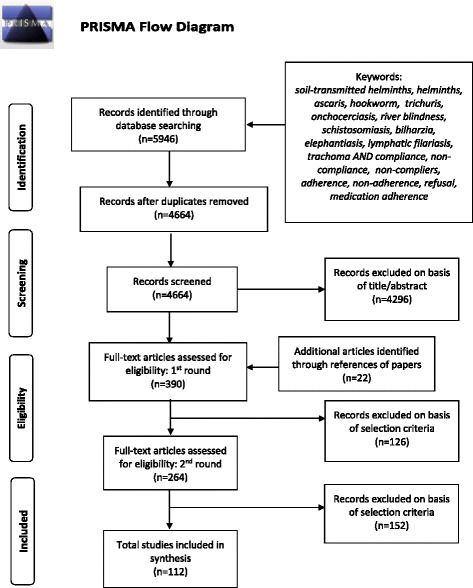
Selection of studies for inclusion in the review. PRISMA flow diagram outlining the literature selection process

The literature selection process is outlined in Fig. 2. The search generated 4664 citations (excluding duplicates). These studies were initially retrieved and reviewed at title and abstract level (consulting the full text if no abstract was included). In the second round, 368 articles were read in full and assessed for eligibility. A further22 papers were identified from reference lists of other papers (totalling 390 for review). An additional round of full-text review was completed on 264 papers, and a total of 112 papers were eventually included in the final count.

A data extraction form was developed in Microsoft Excel and included four major components: compliance/non-compliance rate, reasons given for low/non-compliance, whether the study included statistical analysis of factors associated with compliance/non-compliance, as well as notes on how compliance was defined in the studies. Due to the wide range and heterogeneity of the factors associated with compliance found in the literature, these data were not reported in full. Other details of the publication and PCT were also included (see Table 2 for summary; full data table in Additional file 3).

**Table 2:** Subset of data extraction table –five of 112 studies shown (full table in Additional file 3)

Reference	Publication Year	Country	MDA Year(s)	Disease	Drugs delivered^a^	Method of delivery	Sample^b^	Compliance rate	Reasons for non-compliance	Predictors of compliance (y/n)^c^	Notes on metrics/definitions
[31]	2008	Nigeria	1996–2004	RB	IVM	Community	4800 surveyed	49.96 % overall participation	n/a	No	Presented as ‘coverage’ yet discussed as ‘participation’
[32]	2007	Sri Lanka	2003	LF	DEC + ALB	Community	4358 surveyed	71.4 %	Taking other medication (3.1 %), felt they did not need them (3.2 %), had forgotten to consume them (1.1 %), worried about adverse effects (0.8 %)	No	Compliance: those who consumed drug over eligible population
[34]	2013	India	2008	LF	DEC	Community	571 eligible	42.3 %	No motivation (24.7 %), drugs not supplied (22.5 %), absence at home (13.5 %), no faith (10.1 %), fear of side effects (10.1 %) and others: Forgotten, lack of prior IEC etc. (7.8 %), illness (7.3 %), wrong information (3.9 %)	No	Compliance: consumption of drug among those who received drug; defaulter: did not consume drug, or partially consumed drug, or those who were not supplied the drug by the drug distributors
[30]	1991	Liberia	1987–1989	RB	IVM	Community	1987: 13,704; 1988: 13,977; 1989: 14,110	1987: 96.8 % (56.2 % of total pop); 1988: 96.6 % (57.7 % of total pop); 1989: 98.4 % (70.9 % of total pop)	n/a	No	Referred to as ‘those who accepted treatment’ using eligible population as denominator
[53]	2006	India	2001–2003	LF	DEC/DEC + ALB	Community	unspecified	MDA rounds 1–3 consumption rate: 34.9 %, 39.8 %, 41.7 % (of total population); 35.5 %, 40.3 %, 42.4 % (of eligible); 46.9 %, 51.7 %, 50 % (of drug recipients)	MDA rounds 1–3: not necessary (31.8 %, 52.9 %, 42.9 %), fear of side reactions (24.6 %, 20.7 %, 30.1 %), treatment for other diseases (8.9 %, 4.4 %, 11.3 %), no opinion/no response (19.2 %, 2.5 %, 6.5 %), partial consumption (6.6 %, 4.4 %, 5.6 %), others (8.9 %, 15.1 %, 3.6 %)	No	Referred to as consumption rate and presented in terms of total population, of eligible population, and of those who received drug

^a^some studies may involve combinations of drugs (i.e. DEC + ALB) yet refer to only one drug (i.e. DEC) in the paper; the table includes only those drugs specifically named in the studies

^b^when available, the surveyed/interviewed population was taken as the sample; otherwise, eligible or total study population was taken

^c^only when statistically supported

### Data synthesis and definitions

For the purpose of this paper, the authors’ use of the term ‘compliance’ refers to the proportion of individuals who swallow the medications (whereas ‘coverage’ refers purely to drug delivery: the proportion of individuals who receive the drug). Due to the heterogeneity of definitions and terms used through the collection of studies, it is important to note that the denominators used are, in some papers the total population, and in others the eligible population. These issues will be addressed further in the discussion.

As one of the main themes of the paper is to highlight the inconsistencies in definitions and calculations of compliance, the data are reported as they were in the original publication since it was not possible to re-analyse or standardise the data in all studies. At times, the calculation of compliance data or statistics associated with compliance-related factors were unclear. However, the authors did their best to accurately represent the results given the available information.

## Results

A total of 112 studies met the criteria for inclusion in the review (Fig. 2). The majority of the studies were selected through the search of databases, but some additional relevant studies (22) were identified through references listed in the selected papers.

### Characteristics of included studies

**Fig. 3:**
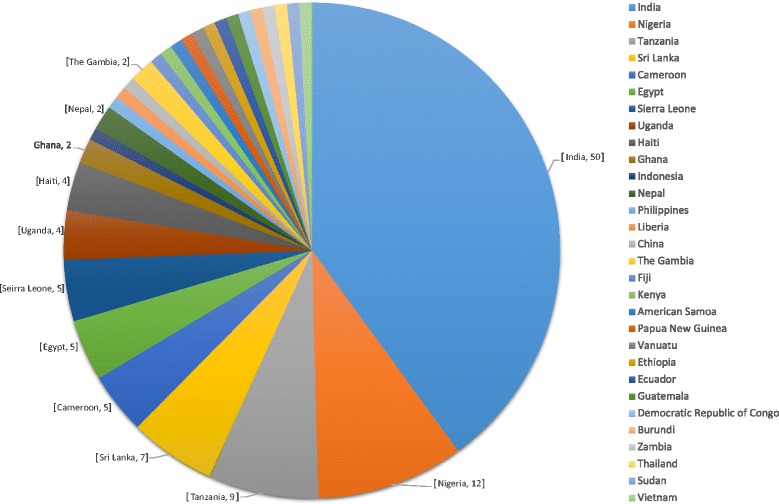
Number of publications by country. Unlabelled segments of the chart represent one study for the respective country. For publications involving multi-country studies, each country was counted towards the total

**Fig. 4:**
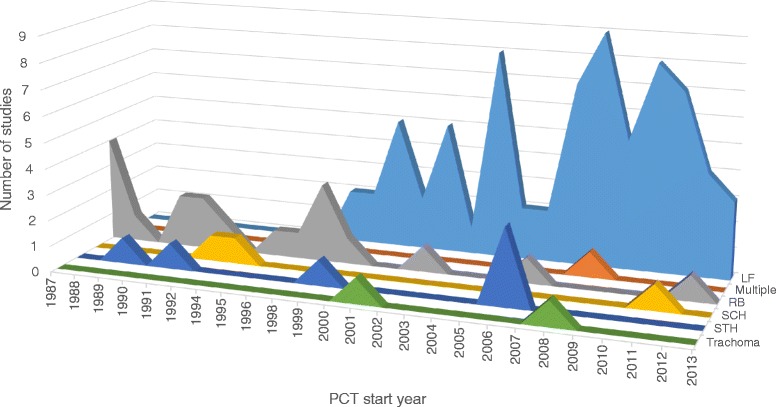
Number of studies by MDA start year. The studies include both single-round and longitudinal studies

**Fig. 5:**
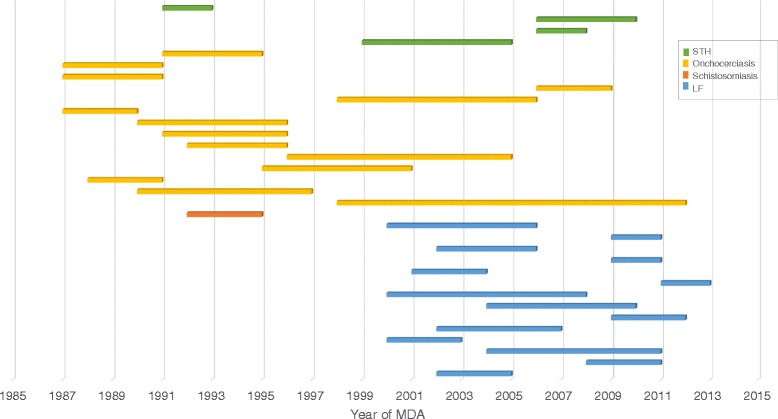
Duration of longitudinal studies by disease. The studies include those for which the PCT start and end year were available

**Fig. 6:**
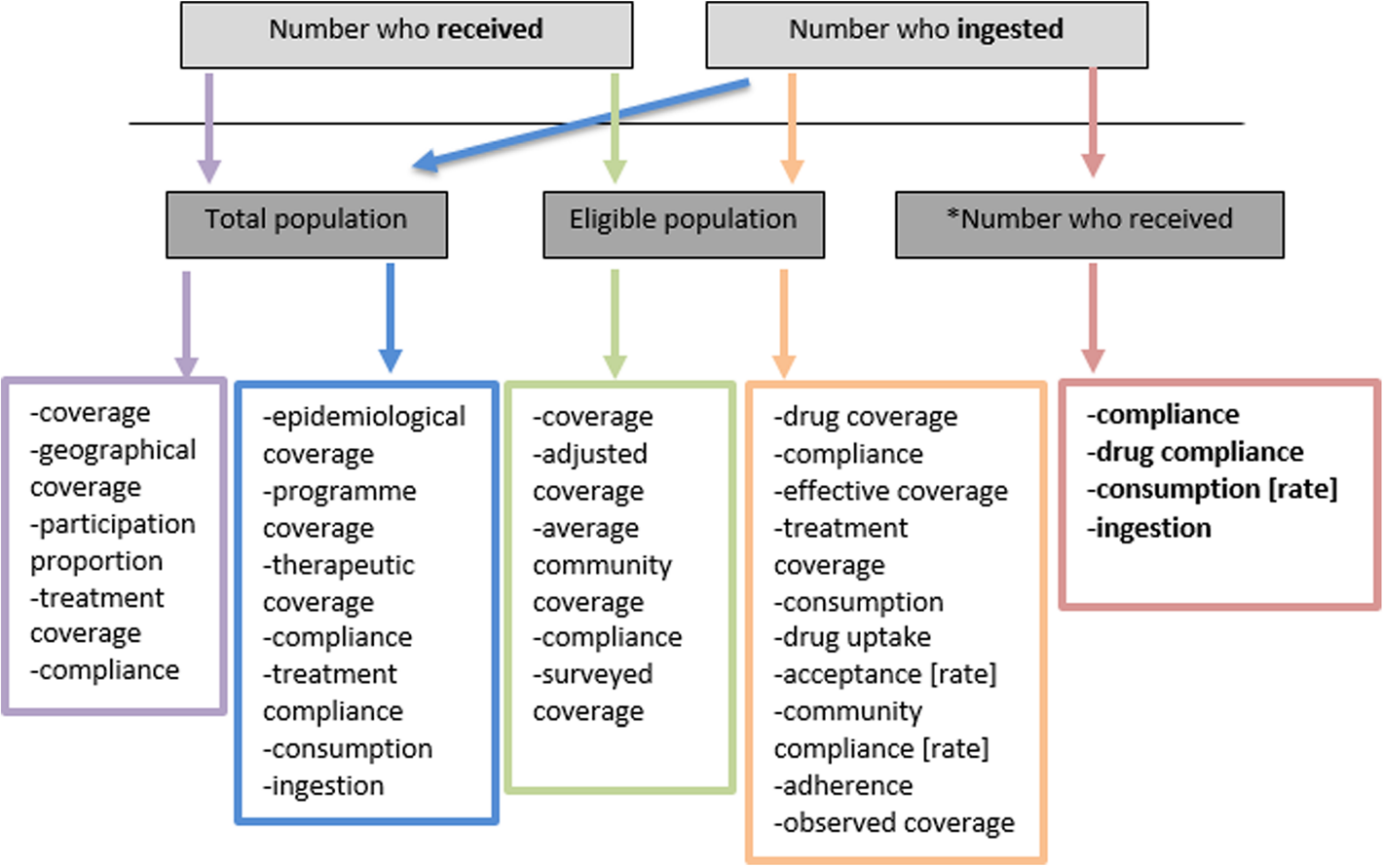
Combinations of numerator/denominator and respective terms employed in the selection of studies. The coloured arrows and boxes represent the various combinations and resulting terms that researchers have employed in the calculation of compliance, or other ‘medication-taking’ terms.*‘Number who received’ represents the most “selective” denominator. The bolded terms resulting from this metric (number who ingested/number who received) therefore capture the most accurate measure of compliance

Details of the selection of papers are shown in Additional file 3. The study designs of the selected papers included cross-sectional studies, intervention studies, coverage surveys of various types, and household surveys, with use of both self-reported questionnaires and directly-observed treatment. The search retrieved 36 longitudinal studies (reporting more than one round of PCT) accounting for 32 % of the total studies. The papers reported on evaluations of PCT/MDA rounds carried out from 1987 to 2013 in 30 countries with almost half the studies coming from India (50). The full breakdown of studies by country is shown in Fig. 3. The number of studies by disease and year are shown in Fig. 4 for all studies, and in Fig. 5 for multiple-round/longitudinal studies only. In terms of method of delivery, the majority of studies were community-based or -directed PCT (including central distribution points) as compared to school-based studies which were only three. The identified studies were very heterogeneous in terms of outcome variables (see Fig. 6 for details).

### Compliance rates and metrics

The selection of studies revealed a wide range of compliance data. The rates presented ranged from 19.5 to 99 % compliance (or a similar metric). There was, however, considerable variation in the terminology and definitions used. Due to such heterogeneity, it was not possible to comparatively assess the data, especially when authors fail to define the terms or metrics used in their analysis. Approximately a quarter of the studies in the review were classified as having unclear compliance data—meaning the metric or denominators used were not defined and unable to be inferred from the paper. A further set of studies (40) were previously excluded due to even greater degrees of ambiguity regarding definitions and calculations.

### Longitudinal studies

There were a total of 36 selected studies which assessed more than one round of PCT/MDA with the majority coming from the LF (17) and onchocerciasis (14) literature. These studies are shown by PCT duration and disease in Fig. 5 (three of the 36 studies were excluded in the figure due to unspecified programme dates). The duration of PCT covered in the selection of studies ranged from one year (using bi-annual treatment) to 14 years. Despite reporting compliance rates over multiple time points/rounds of PCT, the majority of these studies assessed compliance retrospectively and not annually at each PCT round. Specific examples and the implications of this methodology are discussed further in subsequent sections.

### Reasons for and factors associated with low/non-compliance

Approximately 56 % (63/112) of the selected studies reported quantitative data on reasons for non-compliance/non-consumption (i.e. percentage distribution). Other studies provided only qualitative or anecdotal data on reasons for low or non-compliance, and these papers were excluded from the review. The studies revealed a range of reasons given for non-compliance or non-consumption including programme-level issues and individual-level characteristics. The former included comments relating to the methods of delivery or distribution, the motivation and/or perception of drugs distributors or health staff, the availability of drugs, the use of directly-observed or supervised therapy. Individual-level characteristics ranged from awareness and knowledge of the disease or PCT programme, perception of risk and benefit of PCT, and drug-related concerns such as forgetting to take the drug, feeling the drug is unnecessary, general dislike of taking drugs/swallowing tablets, fear of taking drugs when ill, and most notably, fear of side effects or adverse events. Fear of side effects (often times based on previous experience of side effects) was among the reasons for non-compliance with MDA for the majority of LF and onchocerciasis studies which used combinations of albendazole (ALB), diethylcarbamazine (DEC), and/or ivermectin (IVM). However, these were also the disease areas which more commonly reported such data; whereas, the selected schistosomiasis, STH, and trachoma papers rarely included assessments of reasons for non-compliance. Reported side effects/adverse reactions from IVM commonly included itching, rash, body pain, fever, headache, swellings, dizziness, and weakness [29–31]. For DEC and ALB regimens, participants reported fever, headache, giddiness, nausea, vomiting, diarrhoea, sleepiness, fatigue, swelling, and myalgia [32–34].

Thirty-one percent (35/112) of the selected studies included statistically-supported data on factors associated with compliance (or with different types of ‘coverage’). These studies involved univariate or multivariate analyses of demographic variables (such as age, gender, education, religion, ownership of land, etc.) and/or individual characteristics or behavioural factors (such as perceived risks and benefits of the disease or PCT programme, knowledge of disease transmission, prevention or treatment, type of recruitment or drug distributor, etc.). As would be expected, these factors show considerable overlap with reasons given for non-compliance; for this reason and due to previous comprehensive reviews on the subject [25], it was decided that this component of compliance is beyond the scope of the current paper, and only the presence or absence of this data was recorded for each study.

#### Interpretation of the data presented in the selected publications

The findings of this systematic review revealed substantial heterogeneity across compliance terms and definitions; an imbalance of available studies for particular disease areas and countries, with a much higher concentration of studies on LF and onchocerciasis and based in India; and finally, a lack of longitudinal compliance studies to properly investigate the role of systematic non-compliance. Most striking was the limited number of compliance studies—and specifically of longitudinal cohort-based studies (following individuals over time)—for schistosome infections (three total; one longitudinal) and STH (six total; four longitudinal) where PCT coverage has increased significantly in recent years [35, 36]. In addition, we found very few studies with school-based or mixed (community + school) delivery with three papers each.

### Compliance terminology and definitions

It is important to be aware of the widespread heterogeneity of coverage versus compliance definitions and calculations throughout the literature. A significant number of papers report ‘coverage’ rates while actually representing ‘compliance’—calculating those who ingest medication and not just receive it, for example—but then referring to it as ‘coverage’, or vice versa. For example, a study states that a high level of *compliance* to the drug was achieved, yet then refers to the percentage of those who *received* the drug (not ingested). In some studies, it seems this variation in terminology may be due to an intervention design of directly observed/supervised treatment. Therefore, when reporting ‘coverage’ rates, one is assuming the consumption of the drug was ~100 %, as the process was directly observed. In this scenario, one may argue there is less of a need to distinguish between receiving and consuming, and between coverage and compliance. This was often the case for many onchocerciasis studies (and some LF studies) which likely followed the African Programme for Onchocerciasis Control (APOC) model of Community-Directed Treatment with Ivermectin (CDTI) which specifies directly-observed treatment (DOT). However, for other studies, this was not clear—the treatment distribution and recording process was not well described and it was not possible to assign studies to a DOT category. Our argument for better distinction between metrics is aimed rather at studies where treatment was not directly observed, or studies where it was impossible to know whether treatment was observed or not. Nevertheless, the type of distribution (e.g. DOT) and the metrics used should be well defined and clarified in the reporting of programme data. It is important to note, that in other areas of repeated chemotherapy (such as for HIV and non-infectious conditions like high blood pressure or other chronic illnesses), modern methods of measuring compliance, and the difference between receipt of the drug and consumption of the drug, are well documented [37–41].

Due to such variations in the definitions for compliance used in the papers, it was challenging to make any comparisons of the data. A true comparative assessment cannot be done across studies which use different definitions or equations of compliance, especially when the authors fail to even define the metric used. A meta-analysis would, therefore, be unrealistic. Krentel et al*.* discuss similar issues of heterogeneity of terms and definitions; they emphasise the importance of distinguishing between coverage (defined as “delivery of medicine”) and compliance (defined as “ingestion of pills”). The authors further illustrate the importance of this distinction when considering the simulation model LYMFASIM, which estimates the impact of PCT on infection and transmission rates by using as a key input “the fraction of people treated per round” [42]. According to the WHO, this calculation is equivalent to ‘epidemiological drug coverage’ (see Table 3) and captures those who actually swallow the drug while ‘reported coverage’ may not include this specification [25, 43]. Krentel et al*.* argues that differences in these two metrics may explain in some part the persistence of LF transmission after multiple rounds of PCT in some countries despite high reported coverage rates [25]. Table 3 highlights the varying types of ‘coverage’ reported in PCT evaluations which are commonly used to convey the concept of medication-taking. These definitions are primarily based on the GPELF guidelines; it is important to note that these terms are also employed outside the LF literature, but they are often used inconsistently in both contexts.

**Table 3:** Various types of coverage terms used in PCT evaluations and definitions based on GPELF (Global Programme to Eliminate LF)

Coverage term	Definition	References and comments
Reported coverage	*“Intervention coverage calculated from data reported by all drug distributors”*	[43]; often much higher than compliance/drug coverage/survey coverage
Programme coverage/ epidemiological drug coverage	*“Proportion of individuals in the implementation unit who have ingested the MDA drugs of the total population in the implementation unit”*	[43]; denominator is total population
Drug coverage	*“Proportion of individuals, expressed as a percentage, in a targeted population who swallowed a drug, or a combination of drugs”*	[43]; denominator is eligible/targeted population
Surveyed coverage	*“Total number of individuals identified by household survey as having ingested the drugs over the total number of individuals residing in all the surveyed households about whom information on drug ingestion could be elicited”*	Relies on self-reporting by participants; subject to recall bias or participants’ assumptions about correct answers to give [25, 43]
Geographical coverage	*“Proportion of administrative units that are implementing MDA of all those that require MDA.”*	[43]; focus on distribution rather than ingestion

Adapted from [25]

### Compliance/coverage denominators

Perhaps the most important component of coverage and compliance calculations (which varies by programme and study) is the denominator. Figure 6 shows the different combinations of compliance (or medication-taking terms) used in the selection of studies. Some studies counted as their numerator the number of people receiving the drug (i.e. coverage); others specified the number of people ingesting/swallowing the drug; while others failed to specify or used ambiguous terms such as ‘number participated’ or ‘number treated.’ To calculate the rate of medication-taking, researchers generally divided this data by: 1) the total population, 2) the eligible population, 3) the number of people who received the drug, or oftentimes, 4) an unspecified denominator. The coloured arrows and boxes represent the various combinations and resulting terms that researchers have employed for that calculation. It is important to note that some studies calculated compliance rates using the total surveyed population (through household questionnaires, for example) as the denominator which is a logical and accurate representation of the compliance given the data collected. Nevertheless, it is clear that the term ‘compliance’ is used (often inaccurately) for different combinations of numerator and denominator—as it appears in all boxes.

Alexander (2015) [26] comments on the difference in using eligible population (drug coverage) versus total population (epidemiological drug coverage) as denominators—the latter of which corresponds to the original LF modelling study [14] establishing the 65 % minimum effective coverage for elimination. He warns against the potential confusion and misreporting of coverage/compliance with regards to Transmission Assessment Surveys (TAS) methodology, suggesting that progress could be overestimated and TAS could be done prematurely if not based on GPELF’s ‘epidemiological drug coverage’ [26]. In this review, we observed a considerable number of LF (and some onchocerciasis) studies which shared a uniform set of definitions and metrics. This is undoubtedly due to the presence of GPELF definitions and guidelines (Table 3). However, the overall selection of studies across multiple diseases represented a largely heterogeneous set of compliance outputs and definitions as evident in Fig. 6.

### Ideal compliance metrics

Koroma and colleagues state that the best measure of how well PCT programmes are implemented is the ‘drug coverage’—number who ingested the drug over the targeted or eligible population—and that it should be close to 100 %; whereas an adequate level of ‘epidemiological drug coverage’ (using total population as the denominator) is estimated to be 80 % [44]. In their review, Babu and Babu focus on the coverage-compliance gap (i.e. the difference between those who receive the drug and those who actually consume the drug) as the primary metric for capturing the proportion of people who receive but do not ingest. Over a total of 36 studies, coverage ranged from 48.8 to 98.8 % whereas compliance was, on average, 22 % lower [17]. This is probably the most ideal and useful measure of true ‘compliance’ but required the authors to re-calculate many of the papers’ compliance rates so as to have consistent denominators and a uniform metric for comparison. In their recalculations, they chose what some of the research community refers to as ‘effective coverage’ as their standard metric for comparison—reporting those who ingested the drug over the eligible population.

Considering the variation in terminology, one could argue that each definition or calculation may have its place in representing different aspects of coverage and compliance—useful at different levels of programme design and evaluation. For example, using *total* population as the denominator may have greater epidemiological value in showing the proportion of those at risk being covered by PCT, whereas employing *eligible* population may be more valuable in assessing the effectiveness of the programme. Finally, using the most selective denominator of drug *recipients* provides a more individual level of acceptability and compliance—enabling researchers to capture those who choose *not* to take the drug but only if they have been offered it in the first place. This is a much more accurate representation of drug refusal and would better inform programme design and implementation—and more specifically, in the context of community sensitisation.

The lack of consistency among reporting of compliance data, however, can result in under- or over-estimating compliance in a population, and therefore has serious implications for setting and reaching elimination targets. The authors would support Krentel’s recommendations for the research community and national programmes to agree on a uniform set of definitions, but also a standardised method of reporting compliance. As Krentel et al*.* point out, the WHO guidelines [43] are a starting point (summarised in Table 3); evidence of this consistency in definitions is seen in some of the onchocerciasis and LF literature as many studies report comparable rates of both compliance and multiple types of coverage. However, the authors would argue for greater clarification of the distinction between various forms of coverage and compliance to improve understanding. There should be much greater focus on the standardisation of terms and definitions so as to facilitate reliable comparative studies.

### Ideal study design

**Fig. 7:**
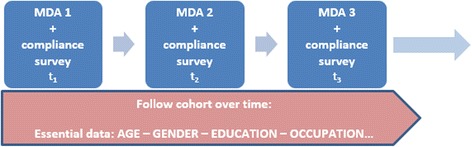
Ideal study design for investigating systematic non-compliance. The figure represents a longitudinal cohort study, following the same individuals over time, stratified by a set of socio-demographic factors. Compliance surveys should be conducted following each treatment round to provide a minimum of three time points

In addition to employing the correct compliance metrics, it is of equal importance to consider the best study design for investigating systematic non-compliance in ongoing control programmes. There is a need for longitudinal cohort studies, following the same individuals over time, stratified by a set of socio-demographic factors (see Fig. 7). There is perhaps a trade-off between the ideal timeline (many years of data) and the need to understand the extent of and reasons for systematic non-compliance quickly. Depending on the frequency of treatment, a couple years or perhaps three time points could be sufficient for getting an initial understanding of the issue, but ideally, data would be captured continuously over the duration of the programme.

Data analysis would aim to quantify ‘predisposition’ by calculating the probability of not complying (through a non-parametric test of correlation between the likelihood of taking the drug at time points t_1_, t_2_, t_3_ etc. using Kendal’s Tau test). An individual’s predisposition could be further categorised into levels of low/medium/high based on relevant socio-demographic or behavioural factors found to influence compliance—which are crucial for a comprehensive understanding of systematic non-compliance.

### Reasons for and factors associated with non-compliance

In addition to the heterogeneity of compliance rates, an unexpected finding from this review was also the heterogeneity in the (reporting of) reasons for non-compliance. For example, some studies include ‘not receiving tablets’ as a reason for non-compliance, whereas others more precisely consider this a reason for non-coverage. In the latter case, only reasons for not consuming/ingesting the drug would be classified as non-compliance. It is also of interest to note that among the reasons given for non-compliance (or non-coverage) were problems with drug delivery (e.g. ‘distributor did not come’ or ‘treatment not supervised’). In this case, considering APOC’s CDTI model of directly-observed treatment, one may argue that what is truly at play here is ‘non-compliance’ or ‘non-adherence’ to treatment guidelines by the part of the drug distributor. This may be an important contribution to future work on understanding the complexities behind compliance and how it’s reported.

Although rarely mentioned by respondents, another reason for non-compliance is already having received treatment by sources other than nationally-run STH control programmes. “Unprogrammed deworming” occurs frequently, and although it may play an important role in the absence of government deworming, it may also overestimate the effectiveness of PCT [45] and has complicated implications on reports of coverage and compliance. This is undoubtedly another crucial area that will require further attention and research.

As previously mentioned, reasons given for low/non-compliance are understandably linked with many of the factors demonstrated to be associated with non-compliance. Similar to Krentel’s findings on LF compliance studies, the factors identified in this review ranged from wider programme and delivery issues to individual recipient demographics and characteristics such as awareness and knowledge, perceived benefits and risks, adverse events, and personal situations [25]. In their assessment of factors related to continuing transmission of LF in Haiti, Boyd et al*.* reflect on the most common reason given for non-compliance which was “Don’t Know”—and question whether it could be a proxy for “Don’t Care” or whether there is some other determinant of compliance that isn’t being captured by the research [46]. There is increasing awareness of the importance in assessing these factors alongside compliance rates (as evidenced by the considerable number of selected studies from all disease categories which reported on these). As previously discussed, further in-depth research of these factors will be a fundamental component of potential investigations into predisposition.

## Systematic non-compliance

There were very few reliable longitudinal studies reported in the literature. Brieger et al*.* comments on the lack of recent large-scale, long-term studies of annual compliance with ivermectin [47], and the authors would add, to a much greater degree for studies of compliance with other drugs and helminth programmes. In the early years of onchocerciasis programmes, there were not enough annual distributions to give useful measures of compliance. However, once the original MDA projects were in operation for over a decade, annual compliance studies became more feasible [18, 48].

The majority of longitudinal studies which aim to asses systematic non-compliance do so through retrospective methodology whereby participants are asked at one point in time about compliance with several previous PCT rounds (or assessed through previous treatment registers) [24, 46, 48–52]. This approach introduces multiple biases—most notably, the challenge of recalling specific PCT rounds over previous years, especially in areas where multiple disease control programmes may be in operation at various times throughout the year. In addition, there is the bias resulting from the people not reached by the surveys (who may also likely be hard to reach during treatment), although they may be counted towards the denominator of the compliance rate. Conversely, those who are not surveyed will not contribute to the numerator of the compliance rate, regardless of their treatment status—a critical detail in data collection and reporting which should be incorporated into programme and study design. Some studies attempt to compare survey responses to treatment registers; however, these were often incomplete and unreliable [53]. Brieger and colleagues found that only 67.2 % of people recalled the same number of treatments received as that recorded in the register; 16.2 % recalled more treatments than recorded while another 16.2 % reported taking IVM fewer times than recorded [49]. As previously discussed, a more rigorous study design would consist of regular surveys conducted immediately following each round of treatment. This was rarely seen in the selected studies, although two examples include Regu et al*.*’s three-year compliance study for LF in Kerala State [54] and Simonsen et al*.*’s assessment of six treatment rounds for LF control in Tanzania [55]. In each study, a selection of participants were interviewed shortly after each MDA to assess individual compliance with the drug.

Individual compliance can be inferred from long-term coverage data; however, as Brieger et al*.* points out, we cannot learn about the factors influencing systematic non-compliance unless there are longitudinal data on the same individuals [47]. Mathieu and colleagues [24] comment on the uncertainty of the effect of systematic non-compliers on elimination in the absence of information about the infectious status of these individuals. It is unlikely, however, that those who systematically fail to comply would have lower infection levels than those who routinely participate in PCT [24]—indeed the converse may apply in many settings (e.g. if predisposed to heavy infection). This is evidenced by studies in Haiti and Egypt, where compared with compliant individuals, those who were systematically non-compliant had higher infection levels [25, 46, 56] and were also less aware that the drugs could prevent LF and that they must be taken yearly [24].

There is an urgent need to better identify, understand, and develop strategies against systematic non-compliance in order to prevent it from jeopardising elimination efforts. The authors would support Kyelem and colleagues’ remarks concerning the need for a better quantitative understanding of the levels of PCT compliance required to interrupt transmission—but perhaps more challengingly, the levels of non-compliance or systematic non-compliance that would still permit elimination [57].

Recently, both Stolk et al*.* and Coffeng et al*.* have discussed modelling approaches for understanding compliance patterns (for onchocerciasis and hookworm, respectively), incorporating the role of a ‘lifelong compliance factor,’ as well as age and gender, into the probability that an individual participates in treatment [58, 59]. Ideally, this factor captures the effect of a range of individual and sociological factors which may influence compliance (such as education, family circumstances, or access to treatment). Using WORMSIM, a generalized individual-based modelling framework for transmission and control of helminths, Coffeng and colleagues illustrated how systematic (non-) participation in PCT with albendazole can considerably reduce the impact of PCT regardless of treatment frequency.

**Fig. 8:**
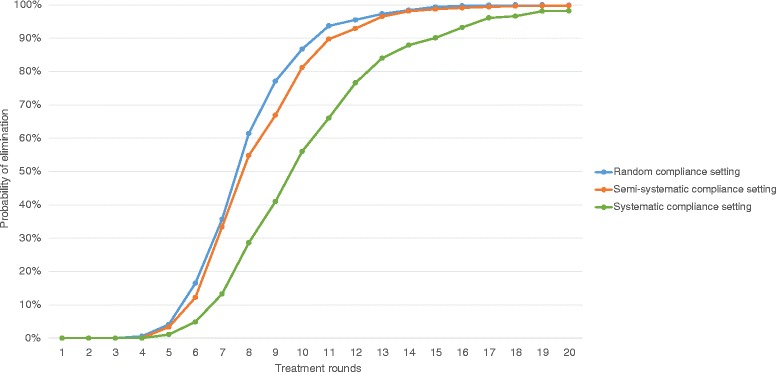
Individual-based stochastic model of STH transmission dynamics and MDA treatment. Elimination curves are shown for different patterns in individual compliance to PCT treatment, targeted at pre-SAC and SAC at 75 % coverage. The blue line represents a random compliance setting where individual participation is randomly allocated at each treatment. The green line represents a fully systematic compliance setting where an individual either always participates (if eligible), or never participates. The orange line represents a semi-systematic (or mixed) compliance setting where some individuals are systematically more likely to participate than others (determined by a lifelong ‘compliance factor’/‘attendance factor’ as termed by Plaisier et al. [60]). Projections are taken from the model presented in [Truscott, J.E., Turner, H.C., Farrell, S.H. and Anderson, R.M. Soil Transmitted Helminths: mathematical models of transmission, the impact of mass drug administration and transmission elimination criteria. Under Review]

Figure 8 shows an alternative approach to modelling STH transmission dynamics which incorporates this lifelong compliance factor and follows a formulation by Plaisier et al*.* [60]. The likelihood of elimination is presented across three compliance settings: random, in which the attending individuals are selected randomly at each treatment; fully systematic, in which individuals either attend all treatments or none; and semi-systematic (a much more plausible scenario), in which individuals attend each treatment according to a lifetime measure of their likelihood to comply (i.e. the compliance or ‘attendance factor’ as Plaisier et al*.* describe it).

In the context of reaching elimination goals, it is also important to consider the potential impact and benefit of alternative treatment strategies such as increasing frequency of treatment (i.e. bi-annual or four-monthly) [8], or using longer lasting drugs such as moxidectin [61]. The authors support previous calls for more and better programmatic data on individual compliance patterns within such scenarios in order to inform the mathematical models used to determine the impact of (non-) compliance on elimination [58, 59] (as models can impact the benefit of changing strategy).

### Limitations

The main limitation of this review in terms of permitting comparative statements and analyses is the level of heterogeneity in both sampling/survey methodology as well as outcome variables (compliance calculations) among the selected studies. Unlike the Babu & Babu review, we did not recalculate the outcome variables to make them comparable across studies, as one of the main objectives of this paper was precisely that—to investigate and highlight the lack of (and need for) such uniformity. The authors purposefully did not apply very strict quality appraisal to the study designs in order to include data from as many endemic areas as possible. This review has taken a fairly collective perspective on PCT compliance in the NTD treatment programmes while focusing less on disease- or programme-specific discussions, as is often the case in the “siloed” research and policy communities. While there are lessons to be learned from the successes and failures of different programmes, there are a set of universal issues and needs to be addressed in all disease areas—especially given the increasing trend for integrating control programmes that target multiple infections.

## Conclusion

Reframing of the guidelines on compliance definitions coupled with an urgent call for longitudinal research in systematic non-compliance should be essential elements in the programmatic shift from control to elimination. With such ambitious goals set out before us, we must first and foremost consider our approach to the data and how we report it, if we are to draw any meaningful conclusions from our research and our programmes. The process of standardisation will be both a source of awareness of the shortcomings in compliance data and research, but it will also be, undoubtedly, the source of greater clarity, progress and successful steps toward elimination. We believe there would be much merit in modifying the current approach for reporting compliance; for example, including fields (with corresponding definitions) for both ‘coverage’ and ‘compliance’ in WHO’s Joint Reporting Form for PCT thereby distinguishing the two metrics and potentially clarifying any discrepancy in language. Having a common digital template to guide the collection of data and its storage on an open access web site is essential. The imprimatur of WHO and major funders of NTD work should continue to facilitate this and to encourage countries to contribute their data. The benefit to the countries is a better understanding of the potential impact on the burden of infection and disease of the PCT programmes they are implementing.

## Additional files

Additional file 1:
**PRISMA checklist.** (PDF 223 kb) Additional file 2:
**full search strategy for PubMed.** (PDF 170 kb) Additional file 3:
**complete data extraction table of all selected studies.** (XLSX 35 kb)

## Abbreviations

(G) PELF: (Global) Programme to Eliminate Lymphatic Filariasis
ALB: albendazole
APOC: African programme for onchocerciasis control
CDTI: community-directed treatment with ivermectin
DEC: diethylcarbamazine
IVM: ivermectin
LF: lymphatic filariasis
MDA: mass drug administration
NTDs: neglected tropical diseases
PCT: preventive chemotherapy
PZQ: praziquantel
RB: river blindness
SCH: schistosomiasis
STH: soil-transmitted helminths
TAS: transmission assessment surveys
