# Supplementary material for: Compliance with anthelmintic treatment in the neglected tropical diseases control programmes: a systematic review
Source: Parasit Vectors. 2016 Jan 27;9:29. doi: 10.1186/s13071-016-1311-1 (PMC4729159; doi:10.1186/s13071-016-1311-1)
Supplement: Additional file 2: — full search strategy for PubMed. (PDF 170 kb) [file 13071_2016_1311_MOESM2_ESM.pdf]

Additional file 2: full search strategy for PubMed

Full search strategy:

("soil-transmitted helminths") OR helminths[MeSH Terms] OR helminths OR helminth OR Helminthiasis OR "intestinal parasites" OR "intestinal helminths" OR ascariasis[MeSH Terms] OR ascari* OR hookworm OR onchocerciasis[MeSH Terms] OR "river blindness" OR schistosomiasis OR schistosomiasis[MeSH Terms] OR bilharz* OR "elephantiasis"[MeSH Terms] OR "lymphatic filariasis" OR "Wuchereria bancrofti" OR "Brugia malayi" OR wucherer* OR brugia* OR strongyloidiasis[MeSH Terms] OR strongyl* OR ancylostomatoidea[MeSH Terms] OR Ancylostoma OR Necator OR trichuris[MeSH Terms] OR trichur* OR whipworm OR geohelminths OR onchocerciasis OR onchocerc* OR trachoma[MeSH Terms] OR trachoma OR trichiasis**)** AND (compliance OR non-compliance OR non-compliers OR adherence OR non-adherence OR refusal OR "Medication Adherence/statistics and numerical data"[MAJR] OR "Medication Adherence"[MAJR])
